# Commentary: Unravelling the effects of additional sex chromosomes on cognition and communication – reflections on Lee et al. (2012)

**DOI:** 10.1111/j.1469-7610.2012.02610.x

**Published:** 2012-10

**Authors:** Dorothy VM Bishop

**Affiliations:** Department of Experimental Psychology, University of OxfordOxford, UK

Most people have 23 pairs of chromosomes; one set from the mother and one from the father. However, nondisjunction errors during meiosis can lead to a case of trisomy, where there are three rather than two chromosomes. Although such events are not uncommon, they are usually lethal, and account for a high proportion of spontaneous abortions. The most common trisomy compatible with survival to birth involves chromosome 21, one of the smallest chromosomes. This is associated with serious developmental abnormalities affecting a range of organs (Down syndrome).

In comparison, the effects of a sex chromosome trisomy are relatively mild. Most children with XXX, XXY or XYY constitutions attend regular school and, apart from a tendency to be somewhat taller than average, appear physically normal. The reason for this can be found in the peculiar nature of the sex chromosomes. The Y chromosome is very small and is thought to contain fewer than 100 genes, compared with over 1000 on the X chromosome. All else being equal, females should have considerably more gene product than males. However, in normal XX females, most of one X-chromosome is inactivated. A process of methylation causes the DNA to be packed in a tight ball, preventing gene expression. This process of ‘dosage compensation’ has the effect of reducing the difference between males and females in amount of gene product.

X-inactivation is not complete; there are regions on the tips of the sex chromosomes that remain active and behave just like autosomes; these are referred to as the pseudoautosomal region. In addition, there are areas of the X-chromosome outside the pseudoautosomal region that escape inactivation, where both copies of a gene are expressed. There appears to be quite substantial individual variation in the number and location of genes that escape inactivation.

If we put these pieces of information together, we start to see why the impact of an additional sex chromosome is relatively mild. For boys with XYY constitution, the additional Y chromosome contains relatively few genes. For girls or boys with an extra X, only one X chromosome is completely activated, and so a minority of X-linked genes are expressed in extra dose.

Nevertheless, there are cognitive impacts of an extra sex chromosome, as discussed in the article by Lee et al. (2012), with intriguing differences between those with an extra X or Y. There is surprisingly little research on sex chromosome trisomies: The explanation is largely due to the mild impact of the trisomy, which means that many people who have a sex chromosome trisomy would not be aware of their status. Most of the information we have about prevalence and consequences of sex chromosome trisomies comes from a set of studies carried out in the 1960–1970s in which newborn babies underwent chromosome screening. Such studies are unlikely to be repeated now for two reasons. First, they are highly labour-intensive: given that, for instance, XXX chromosome constitution is found in around 1 in 1,000 girls, this means we would have to screen 10,000 cases to get a sample of just 10 affected cases. Research is also limited by ethical concerns. The studies done in the 1960s proved problematic as the researchers started to realise that telling parents that their newborn child had a sex chromosome trisomy was bound to cause anxiety and distress, especially since information about its likely impact was so uncertain. Findings from the newborn screening studies were summarized in a systematic review by Leggett, Jacobs, Nation, Scerif, and Bishop (2010), who noted a reduction in IQ in XXX, XXY and XYY groups, with both groups of males showing evidence of disproportionate verbal impairments. Such deficits are assumed to be caused by the excess proteins that arise from supernumerary X- or Y-linked genes that escape inactivation.

The report by Lee et al. makes a unique contribution by extending the study of the impact of supernumerary sex chromosomes to include rare cases of children with four or five sex chromosomes. They demonstrate a clear ‘dosage’ effect, whereby the more chromosomes, the greater the negative impact on IQ and development. Each additional X- or Y-chromosome was associated with a decrease in IQ of around 1 *SD*. Intriguingly, they also found that additional X chromosomes had a greater effect on structural language skills, whereas additional Y chromosomes had disproportionate effect on pragmatic problems and autistic features. It should be noted, however, that those with extra X chromosomes included both girls and boys, whereas the extra-Y chromosome group were all male. Bishop et al. (2011) considered boys and girls separately and found that XXY and XYY boys had similar communication profiles, whereas XXX girls had less evidence of pragmatic impairments.

Findings of autistic features in XYY males is of particular interest. When XYY was first described in the 1960s there was intense media interest in the idea that these were ‘supermales’ with unusual levels of aggression and criminality. An early study by Witkin et al. (1976) put these ideas into perspective. Witkin et al. capitalised on the excellent medical, military and criminal public records available in Denmark, as well as the willingness of a high proportion of the population to cooperate by providing DNA samples to the researchers. By restricting their study to tall men, they were able to identify 12 cases of XYY and 16 cases of XXY in a population of over 4,000 males. They confirmed a higher rate of criminality among XYY males than either XY or XXY males, but noted that the crimes mostly involved property rather than aggressive assaults, and could at least partly be explained in terms of the lower IQ of XYY males relative to XY males. The findings on autistic features by Lee et al. (2012) cast the XYY phenotype in a new light, suggesting that a tendency to get in trouble with the law may be related to deficits in social communication and interpersonal skills, rather than unnatural levels of aggression.

Difficult ethical choices still surround the topic of supernumerary sex chromosomes. In many countries, when an abnormal number of sex chromosomes is identified on prenatal testing, the parents are offered a termination of the pregnancy. The study by Lee et al. (2012) therefore has important clinical implications, as well as theoretical importance, because parental choices can be influenced by what they are told about the likely outcome of the child. As the authors note, we must be cautious in interpreting findings based on cases where an unusual karyotype only comes to light when a child is investigated for behaviour problems. Nevertheless, all recent studies have found evidence of an increase in autistic features in boys with XYY identified prenatally, where ascertainment bias is unlikely to account for associations with developmental difficulties. It is, however, important to emphasise that although there is an increased risk of both structural language problems and autistic features in children with additional sex chromosomes, there is wide individual variation. Some children with trisomies do not have any difficulties, and only a minority merit a diagnosis of autistic disorder (Bishop et al., 2011; Ross et al., 2012).
